# Relationship between size‐specific dose estimates and image quality in computed tomography depending on patient size

**DOI:** 10.1002/acm2.12340

**Published:** 2018-05-04

**Authors:** Hiroki Kawashima, Katsuhiro Ichikawa, Shinsuke Hanaoka, Kosuke Matsubara, Tadanori Takata

**Affiliations:** ^1^ Faculty of Health Sciences Institute of Medical, Pharmaceutical and Health Sciences Kanazawa University Kanazawa Japan; ^2^ Radiology Division Kanazawa University Hospital Kanazawa Japan

**Keywords:** auto exposure control, computed tomography, contrast‐to‐noise ratio, image quality, patient size, size‐specific dose estimates

## Abstract

This study investigates the relationship between contrast‐to‐noise ratio (CNR) and size‐specific dose estimate (SSDE) in computed tomography (CT) depending on patient size. In addition, the relationship to the auto exposure control (AEC) techniques is examined. A tissue‐equivalent material having human‐liver energy dependence is developed and used to evaluate these relationships. Three exposure dose levels (constant CT dose index, constant SSDE, and with AEC) are tested using four different phantom sizes (diameter: 15, 20, 25 and 30 cm) in two different CT scanners (SOMATOM Definition Flash, Siemens, and LightSpeed VCT, GE). The contrast‐to‐noise ratios (CNRs) are measured using the developed phantom. It is found that the CNR increases with decreasing phantom size at constant SSDE, although the increase ratio is smaller than that of the constant CT dose index. This result indicates that the image characteristics differ even when the patient dose received from the CT examination is equivalent for each patient size. In the case of AEC use, the CNR results of the Siemens scanner exhibit a similar trend to those obtained for constant SSDE, for each phantom size. This suggests that the AEC technique that maintains a constant image quality (CARE Dose 4D) for each patient size corresponds well to the image quality obtained for constant SSDE. These findings facilitate further understanding of the relationship between image quality and exposure CT dose depending on patient size.

## 1 INTRODUCTION

Computed tomography (CT) images are indispensable for disease diagnosis and treatment planning; however, concerns exist regarding both dose management and image quality optimization for the CT examination technique. In particular, previous studies have found that both CT dose and image quality are related to patient size, with more x‐ray photons being required for larger patients to achieve typical image quality.^1^, ^2^, ^3^, ^4^, ^5^, ^6^, ^7^, ^8^, ^9^, ^10^

Two CT dose indicators that are widely used are the volume CT dose index (CTDI_vol_) and dose‐length product, which are also employed for national diagnostic reference levels (DRLs). However, CTDI_vol_ does not reflect patient size and is a metric of radiation output obtained using a reference phantom rather than patient dose. To obtain the patient dose, the size‐specific dose estimate (SSDE) has been proposed by American Association of Physicists in Medicine (AAPM) Task Groups 204 and 220.^11^, ^12^ In this approach, the patient dose can be estimated by multiplying CTDI_vol_ by conversion coefficients determined with consideration of the patient size.

The image quality also varies in relation to patient size; however, many of the reports that have evaluated this relationship have considered image noise only as the image quality indicator, and utilized a uniform phantom.^5^, ^7^, ^13^, ^14^ This approach was adopted in those studies because the primary goal of CT examination is soft‐tissue evaluation.^15^ The energy dependence of soft tissue, that is, the contrast variation, is extremely small compared with those of contrast media and bone. In addition, no phantom having human‐tissue‐equivalent energy dependence was available to those researchers. Evaluations based on noise ignore the object contrast; thus, this approach is less relevant to image quality, because the contrast affects the lesion detectability.^10^ The soft‐tissue contrast and image noise change slightly with patient size. Therefore, considering its relation to exposure dose, the contrast‐to‐noise ratio (CNR) is an optimal index for image quality.

With regard to related research considering patient size, image quality, and CT dose, Boone et al.^1^ have examined this topic in detail. In the literature, dose reduction protocols to achieve equivalent image quality based on patient size have been proposed. However, to the best of our knowledge, few studies have evaluated the image quality obtained under the equivalent patient dose received during CT examination. Further, as a CT technology related to patient dose and size, auto exposure control (AEC) is one of the most important techniques. AEC strategies are broadly divided into two categories regardless of attenuation level: “constant image noise (noise index‐based technique)” and “constant image quality (reference mAs‐based technique).”^2^, ^3^, ^4^, ^5^, ^6^, ^7^, ^8^, ^9^, ^16^, ^17^ Although these behaviors differ with respect to patient size, the relationship between the SSDE and image quality has not been adequately evaluated.

In this study, we develop a tissue‐equivalent material having the same energy dependence as the human liver and investigate the relationship between the CNR and SSDE depending on patient size. In addition, we examine the relationship between those parameters and the image quality given by AEC.

## 2 MATERIALS AND METHODS

### 2.A Tissue‐equivalent material

A tissue‐equivalent material having the same energy dependence as the human liver was jointly developed with Kyoto Kagaku Corporation (Kyoto, Japan). The inside of a 20‐cm acrylic cylindrical case was filled with water and the phantom was set in place. Here, both the developed phantom and an existing phantom (SZ tissue‐equivalent phantom, Kyoto Kagaku) were used for comparison. The CT numbers were measured at various energies to validate the accuracy using virtual monoenergetic images. Dual‐energy CT (DECT) scans were performed using a dual source CT scanner (SOMATOM Definition Flash; Siemens Healthcare, Erlangen, Germany). The tube voltages were set to 100 and 140 kVp with a tin (Sn) filter. The DECT raw data were then transferred to a workstation (Syngo Multimodality Workplace, Siemens Healthcare), and the monoenergetic images were reconstructed at 10‐keV intervals from 40 to 160 keV using a dedicated application (Monoenergetic; Siemens Healthcare). In addition, the liver CT numbers of 14 patients were measured retrospectively based on clinical data obtained through dual‐energy scanning of the abdomen. The use of patient data was approved by the ethics committee of our institution.

**Figure 1:**
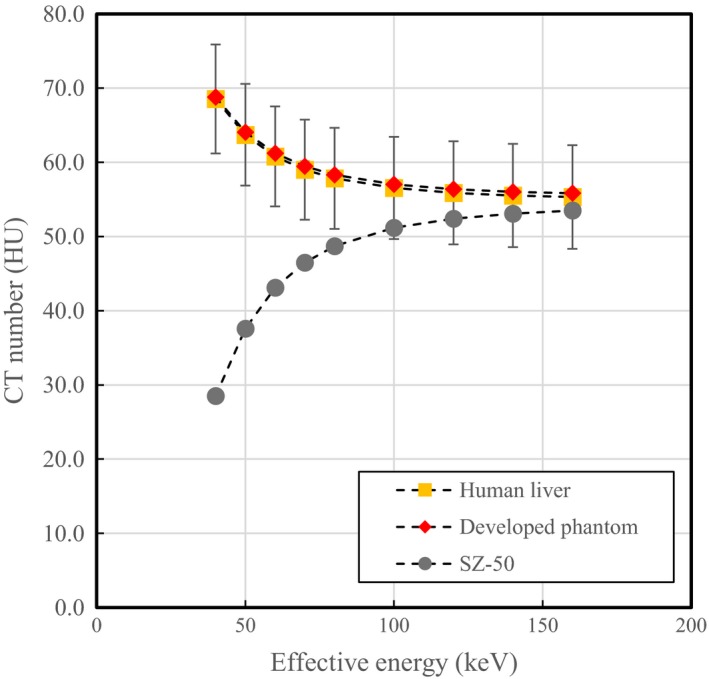
Relationship between CT numbers and energy for virtual monoenergetic images for tissue‐equivalent material (red diamonds), existing phantom (SZ, gray circles), and clinical data (human liver, yellow squares). The CT numbers of the patient livers included individual differences.

Hence, the developed phantom was found to have almost equivalent CT numbers to the patient livers, although the CT numbers for the patient livers included individual differences (Fig. 1). In contrast, the CT numbers of the existing phantom decreased at lower energy; this phantom did not have sufficient energy dependence. Therefore, we decided to use the developed phantom as an object in this experiment.

### 2.B Phantom and scan protocols

**Figure 2:**
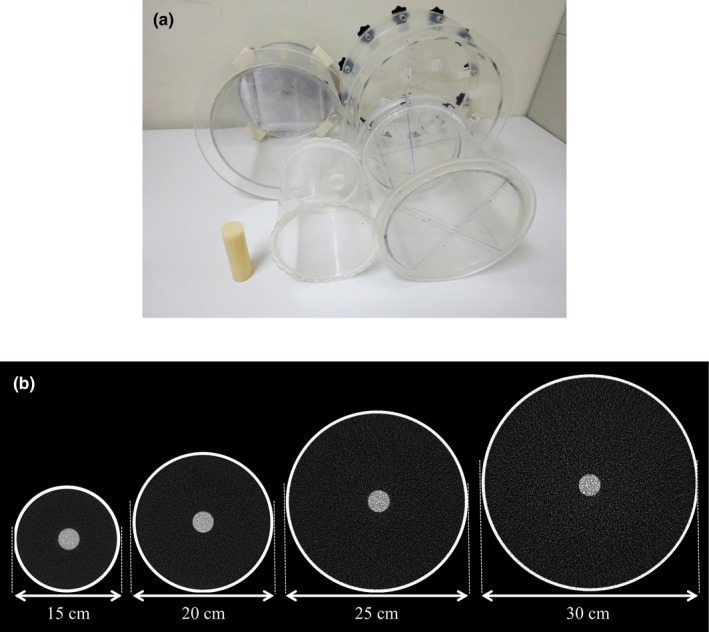
(a) Photograph and (b) axial CT images of cylindrical phantoms used in this work, having 15, 20, 25, and 30 cm diameters. The tissue‐equivalent material was set co‐axially within each case.

We employed four differently sized cylindrical cases having diameters of 15, 20, 25, and 30 cm. The developed tissue‐equivalent material (3.0 cm diameter) was set at the center of each differently sized case, and the case was filled with water (Fig. 2). For each case size, the phantom was placed at the center and imaged using two different CT scanners equipped with different AEC techniques: a 128‐slice multi detector CT (SOMATOM Definition Flash; Siemens Healthcare) and a 64‐section CT (LightSpeed VCT; GE Healthcare, Milwaukee, WI, USA). The image protocol was that used for standard abdominal examination in our hospital. The scan parameter details are listed in Table 1.

**Table 1:** Imaging settings of each CT scanner

	Definition Flash	LightSpeed VCT
Detector row	128	64
Tube voltage (kV)	120	120
Slice thickness (mm)	3	2.5
Filter kernel	D40	Standard
Detector configuration	128 × 0.6	64 × 0.625
Pitch	0.6	0.513

Three dose levels were tested for each scanner to assess mutual relations. First, the CTDI_vol_ value displayed on the console was fixed at 20 mGy regardless of phantom size (constant CTDI). This CTDI_vol_ value was chosen based on the Japanese *DRLs* 2015 for an abdominal/pelvic CT examination.^18^

Second, the SSDE was fixed at a constant level. The SSDE is defined as follows:

(1) $$SSDE=f_{\mathrm{size}}^{32X}\times CTDI_{\mathrm{vol}}^{32}$$

where *f* is the conversion factor obtained as a function of the patient's effective diameter. The *f* values for the SSDE are listed in Table 1D of AAPM Report 204.^11^ For a CTDI_vol_ of 20 mGy and a 30‐cm‐diameter phantom, SSDE was calculated as 24.6 mGy. We employed this dose level as a reference. The image settings were adjusted by changing the tube current to set the CTDI_vol_ value for each phantom size, as detailed in Table 2.

**Table 2:** CTDI_vol_ values and conversion factors to yield constant SSDE for each phantom size

Phantom size (cm)	CDTI_vol_ (mGy)	Conversion factor	SSDE (mGy)
15	11.49	2.14	24.6
20	13.82	1.78	24.6
25	16.62	1.48	24.6
30	20	1.23	24.6

Third, the phantom was scanned using CARE Dose 4D (Siemens Healthcare) and Auto mA (GE Healthcare) to evaluate the difference in AEC metrics. For the AEC metrics of the Siemens scanner, CARE Dose 4D adapted the tube current to the individual patient size based on the quality reference mAs. The mA correction factor for patient size was determined by the strength setting of the CARE Dose 4D. The strength, that is, the change in the ratio of tube current modulation relative to a reference tube current based on the attenuation level, was selected from among five options: very weak, weak, average, strong, and very strong, where the latter gave the largest variation. It was necessary to set two strengths for the smaller and larger cases compared with the attenuation level for standard body size. In this study, the image quality reference mAs was set to 500, and the strength was set to weak and very strong for the smaller and larger cases, respectively. For the AEC metric of the GE scanner, the tube current modulation was calculated based on the noise index entered by the operator. The noise index value is approximately equivalent to the SD measured in the central region of the image for a uniform phantom, and a constant noise level is maintained in the image, independent of patient size. In this study, this value was 8 Hounsfield units (HU), and the tube current was set to a mA range of 10−700.

### 2.C Evaluation approach

At each exposure dose level, the CNR was measured for each phantom size. Regions of interest set on the tissue‐equivalent material and next to the water region (background) were used to measure the average CT number and SD. The CNR was calculated from the relation (ROI_m_ − ROI_b_)/SD_b_, where ROI_m_ and ROI_b_ are the CT numbers of the tissue‐equivalent material and background, respectively, and SD_b_ is the standard deviation of the background.

The figure of merit (FOM) was defined as FOM = CNR^2^/Dose.^19^, ^20^, ^21^, ^22^, ^23^, ^24^ The FOM is an indicator used to evaluate image quality, which is normalized for the exposure dose and indicates the dose efficiency. On that basis, the dose ratio required to obtain an FOM comparable to that of the 30‐cm‐diameter phantom was calculated using the CNR as an image quality indicator. The SD was also used as an indicator, ignoring contrast, according to the relation FOM = (1/SD^2^)/Dose. In this study, to demonstrate the effect of this choice, the CNR and SD were both taken as indicators of image quality, and the dose ratio required to obtain the same image quality as that for the 30‐cm phantom was examined.

## 3 RESULTS

**Figure 3:**
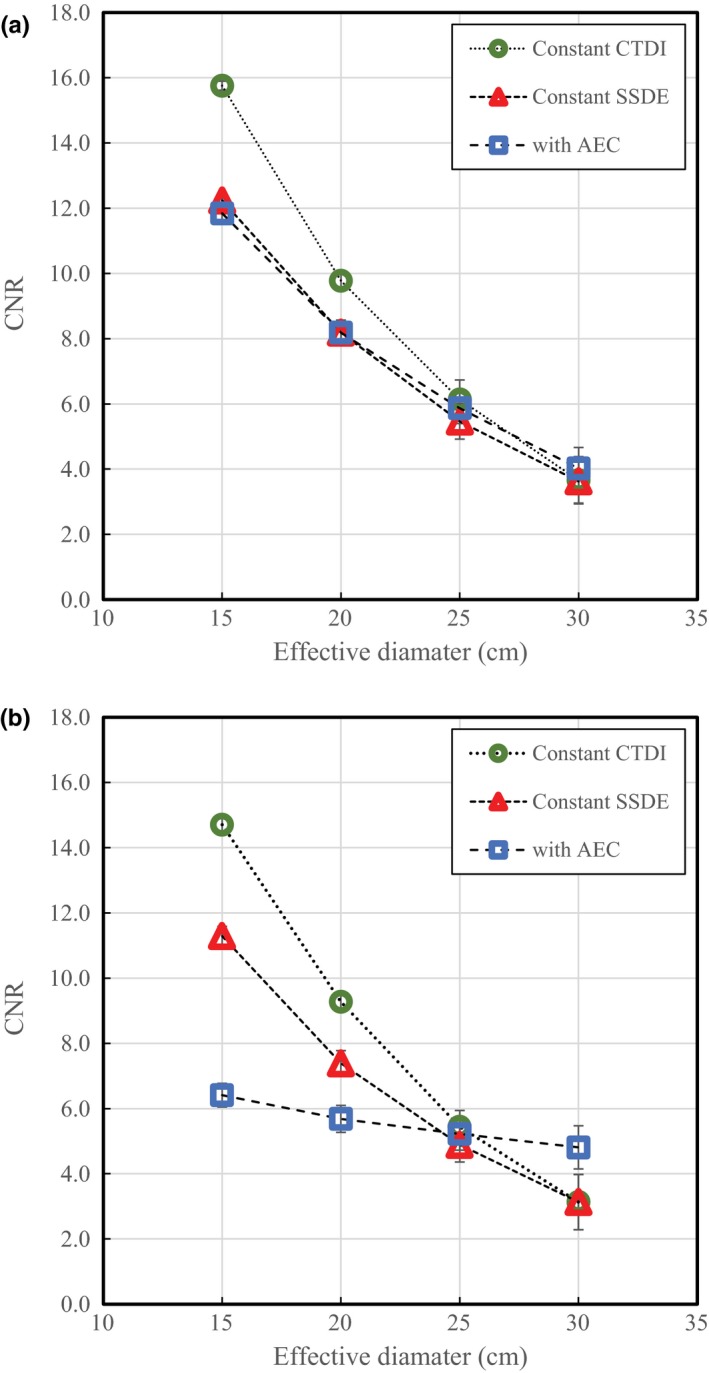
Relationship between CNR and phantom size for a) Siemens and b) GE scanners. Green circles, red triangles, and blue squares: constant CTDI, constant SSDE, and with AEC, respectively.

Figure 3 shows the CNR results yielded by each CT scanner for constant CTDI and SSDE, and with AEC. For constant CTDI, the CNR increased greatly with decreasing phantom size for both CT scanners. The increase ratios for the Siemens and GE scanners (based on the 30‐cm phantom results) were 4.31 and 4.69 for the 15‐cm phantom, 2.67 and 2.96 for the 20‐cm phantom, and 1.68 and 1.74 for the 25‐cm phantom, respectively. For constant SSDE, the CNR also increased as the phantom size decreased, although the increase ratios were smaller than those for the constant CTDI. The increase ratios for the Siemens and GE scanners (based on the 30‐cm phantom results) were 3.38 and 3.60 for the 15‐cm phantom, 2.26 and 2.36 for the 20‐cm phantom, and 1.51 and 1.56 for the 25‐cm phantom, respectively. When AEC was employed, the CNR results yielded by the Siemens scanner exhibited a similar trend to those for constant SSDE for each phantom size. In contrast, the results yielded by the GE scanner differed from those obtained for constant SSDE. In particular, the CNR result for the 15‐cm phantom was 1.33 times higher than that for the 30‐cm phantom (Fig. 3).

**Figure 4:**
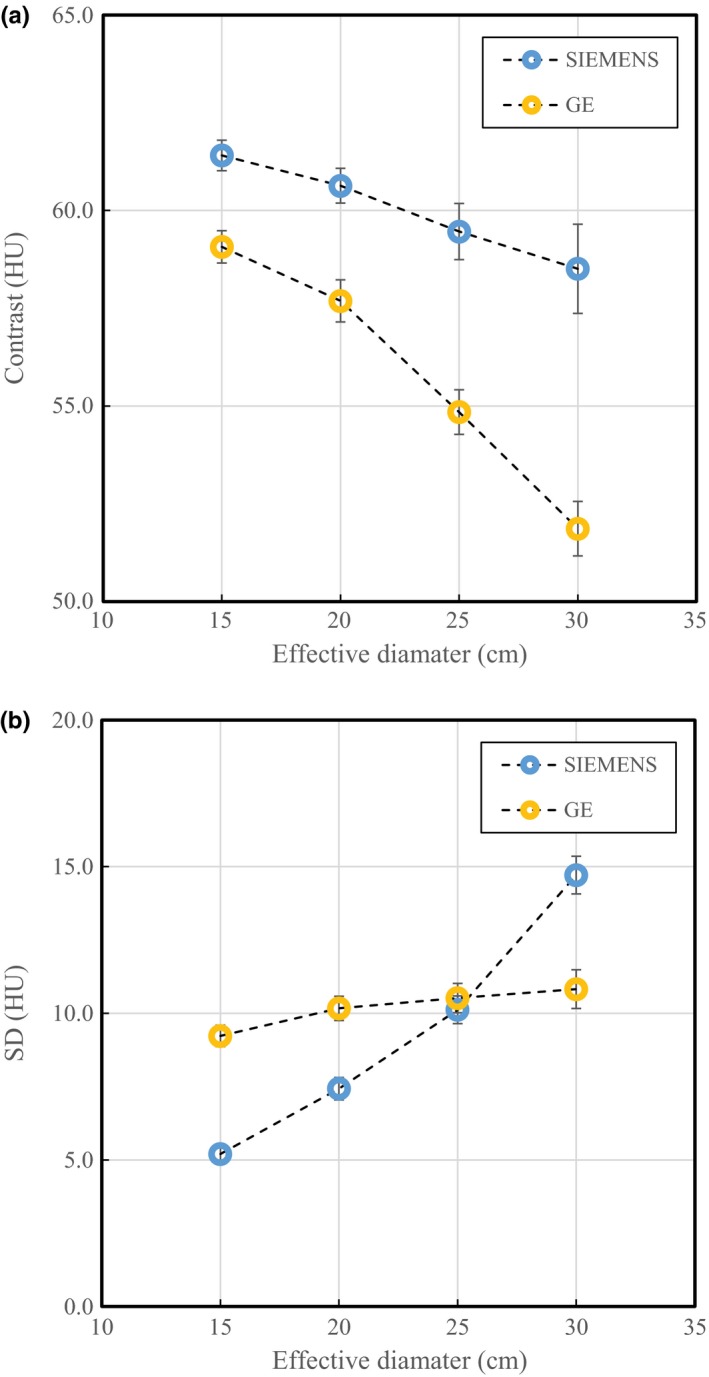
Relationship between phantom size and measured a) CT number of tissue‐equivalent material and b) background noise (SD) for both CT scanners using AEC. Blue and yellow circles: Siemens and GE scanners, respectively.

Figure 4 shows the contrast and background SD as functions of phantom size when the AEC was employed. The contrast results for the Siemens scanner varied from 59.1 HU for the 30‐cm phantom to 61.4 HU for the 15‐cm phantom. Further, the GE results varied from 51.9 HU for the 30‐cm phantom to 59.1 HU for the 15‐cm phantom. The SD results for the Siemens scanner varied in accordance with the phantom size; however, the GE results were almost constant.

**Figure 5:**
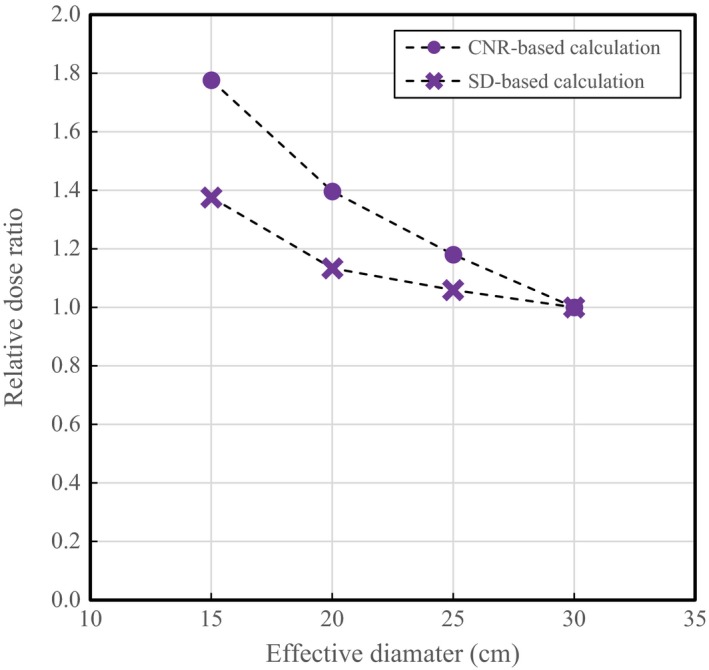
Relationship between phantom size and relative dose ratio to obtain equivalent FOM (normalized to unity at 30 cm) using GE scanner. The CNR (circles) and SD (crosses) are compared as image quality indicators.

Figure 5 shows the relative dose ratios to obtain equivalent image quality (FOM) calculated based on the SD and CNR and as functions of phantom size, with the 30‐cm phantom as a reference and using the GE scanner, for which the contrast variation was increased depending on phantom size. Dose ratios of 1.40 and 1.13 for the 20‐cm phantom and 1.78 and 1.38 for the 15‐cm phantom were obtained using the CNR and SD, respectively. The ratio calculated from the SD clearly decreased in comparison with that obtained from the CNR.

## 4 DISCUSSION

The developed tissue‐equivalent material exhibited energy‐dependent CT numbers similar to those measured for the liver from patient data. Thus, sufficient accuracy was achieved for the phantom to be used as a tissue contrast object for CT examination. We used this material to evaluate the relationship between exposure dose and image quality. The results of this phantom study, which the CNR increased as the phantom size decreased, demonstrate that the image quality evaluation differs significantly depending on patient size, even when the patient dose received from the CT examination is equivalent for each patient size. Considering the impact of phantom size on image quality, a subject size suitable for the purpose of the experiment should be selected in phantom experiments.

Our results show the same tendencies as the relationship between the AEC technique and image quality reported in previous work.^4^, ^5^, ^6^, ^7^, ^8^ The results for “the noise index‐based AEC technique” (Auto mA and LightSpeed VCT, GE Healthcare) indicate that almost constant image quality is maintained regardless of phantom size. On the other hand, for the “reference mAs‐based AEC” (CARE Dose 4D and SOMATOM Definition Flash, Siemens Healthcare), higher CNR was obtained as the phantom size decreased. This is because the latter AEC technique is based on the concept that the same noise levels are not required to obtain adequate image quality for differently sized patients. One of the most curious findings is that the CNR results obtained for constant SSDE and using this AEC technique exhibit a similar trend. However, the results are dependent on the AEC strength, which should be chosen according to the noise level required for a specific diagnostic task.

At maximum contrast change, a difference of 7.2 HU was observed for the GE scanner compared with the Siemens scanner [Fig. 4(a)]. This depends on the effective energy of the x‐ray beam in each scanner. We measured the effective energy in a preliminary study, obtaining 55.3 and 59.0 keV for the GE and Siemens scanners, respectively. Therefore, the contrast was slightly changed by the effect of beam hardening due to different phantom diameters. In particular, the GE scanner with low effective energy exhibited a large variation.

Regarding the relationship between the AEC and image quality, the image noise has been adopted as an indicator of image quality in many studies.^5^, ^7^, ^13^, ^14^ Although the image noise (i.e., SD) can be used to evaluate the variation of the tube current modulation, image quality evaluation ignoring contrast variation due to beam hardening is not appropriate in some cases, considering the relationship between scan parameters and dose in clinical situations. The results of this study demonstrate that the SD‐based dose ratio was underestimated compared with that based on CNR, even if the soft‐tissue contrast was targeted.

Our study has some limitations. First, cylindrical phantoms only were used in the experiments. The human body has an elliptical shape in many cases, and the body shape affects the AEC. Many studies have evaluated the relationship between AEC and patient size by assembling realistic human shapes from those of small to large patients.^1^, ^7^, ^19^ The aim of the present study was to investigate several aspects of the relationship between SSDE and image quality; thus, the effect of body shape was not considered. Second, although the AEC incorporates various user‐controlled parameters such as the noise index, reference mAs, and the strength of the CARE Dose 4D, we did not assess the effects of parameter adjustment. Finally, in clinical CT, low tube voltages, such as 80 and 100 kVp, are selected depending on the aim and patient size. In future research, the effect of the tube voltage on the relationship between the SSDE and image quality should be investigated.

## 5 CONCLUSION

We developed a tissue‐equivalent material having human‐liver energy dependence, which was used to evaluate the relationship between image quality and dose depending on patient size. The results of this study reveal that the image characteristics differ even when the patient dose received from the CT examination is equivalent for each patient size (constant SSDE). In addition, the AEC that retains constant image quality (CARE Dose 4D), rather than constant noise level (Auto mA), for each patient size corresponds well to the image quality obtained for constant SSDE. These findings facilitate further understanding of the relationship between image quality and exposure CT dose depending on patient size.

## CONFLICT OF INTEREST

The authors declare no conflict of interest.
